# Clinical Significance of miR-4535 and miR-191-5p in Maternal Serum as Independent Biomarkers for Severe Chorioamnionitis

**DOI:** 10.7759/cureus.72120

**Published:** 2024-10-22

**Authors:** Koko Ishida, Chihiro Kiyoshima, Daichi Urushiyama, Toyofumi Hirakawa, Shiori Imi, Makoto Hamasaki, Shinichiro Nagamitsu, Makoto Nomiyama, Kenichiro Hata, Fusanori Yotsumoto

**Affiliations:** 1 Department of Obstetrics and Gynecology, Faculty of Medicine, Fukuoka University, Fukuoka, JPN; 2 Department of Pathology, Faculty of Medicine, Fukuoka University, Fukuoka, JPN; 3 Department of Pediatrics, School of Medicine, Fukuoka University, Fukuoka, JPN; 4 Department of Obstetrics and Gynecology, National Hospital Organization Saga Hospital, Saga, JPN; 5 Department of Maternal-Fetal Biology, National Research Institute for Child Health and Development, Tokyo, JPN

**Keywords:** amniotic fluid, chorioamnionitis, fetal infection, mir-191-5p, mir-4353

## Abstract

Introduction: Chorioamnionitis, a perinatal condition caused by fetal membrane inflammation, results in preterm birth, neonatal sepsis, necrotizing enterocolitis, and brain disease in infants. However, predicting maternal and fetal prognoses is challenging. We aimed to assess the relationship between fetal infection induced by severe chorioamnionitis or morbidity and the expression levels　of serum miR-4535, miR-1915-5p, and miR-191-5p levels, which are promising biomarkers for chorioamnionitis, in pregnant women with chorioamnionitis.

Methods:We collectedserum and amniotic fluid samples from 40 pregnant women with preterm labor and analyzed miR-4535, miR-1915-5p, and miR-191-5p expressions. We calculated the area under the curve (AUC) and Youden index to examine the diagnostic accuracy of infection-induced fetal morbidity.

Results: The serum miR-4535 and miR-191-5p levels were significantly higher in patients with severe chorioamnionitis than in those with chorionitis or sub-chorionitis (*P* = 0.001 and 0.003, respectively). The AUC of miR-4535 and miR-191-5p (0.864 and 0.836, respectively) indicated their good diagnostic accuracy for severe chorioamnionitis. Significant correlations were observed between serum and amniotic fluid miR-4535 expression (*P* = 0.011) and serum miR-4535 and miR-191-5p expressions. miR-4535 AUC accurately predicted elevated neonatal immunoglobulin M level (AUC = 0.922) and infection-induced fetal morbidity (AUC = 0.805).

Conclusion: Serum miR-4535 and miR-191-5p are associated with infection-induced severe chorioamnionitis and fetal morbidity and maternal infection, respectively.

## Introduction

Chorioamnionitis is a perinatal condition caused by fetal membrane inflammation [1], resulting in preterm birth, neonatal sepsis, necrotizing enterocolitis, and brain disease in infants [2]. Histological diagnosis of chorioamnionitis is based on neutrophil infiltration depth within placental tissues after delivery, known as Blanc’s classification [1]. Given that it is not feasible to diagnose placental infection before delivery, chorioamnionitis diagnosis is often based on the presence of maternal indicators, including fever, tachycardia, leukocytosis, uterine pain, and foul-smelling uterine discharge, and fetal indicators, such as tachycardia [3]. However, predicting maternal and fetal prognoses based on the currently limited diagnostic approach for chorioamnionitis is difficult [4,5]. Early prediction of chorioamnionitis during pregnancy will enable prompt referral of a patient for antimicrobial therapy or pregnancy termination. Therefore, accurate and easy-to-use diagnostic examinations are required to predict severe chorioamnionitis.

MicroRNAs (miRNAs) are small non-coding RNAs (21-25 units) implicated in various cellular processes, including cell proliferation, differentiation, and death, through the transcriptional regulation of target genes [6]. As nanosized vesicular particles, miRNAs are present in diverse intracavitary fluids, including abdominal, pulmonary, and amniotic fluids, and body fluids in circulation, such as plasma, serum, urine, semen, and menstrual blood [7]. Certain miRNAs in biofluids are recognized as ideal biomarkers for systemic illnesses, such as infectious diseases and cancers [7]. Comprehensive microRNA array and molecular biological analyses of the amniotic fluid have indicated that miR-4535 and miR-1915-5p may serve as promising biomarkers for severe chorioamnionitis [8]. In addition, miR-4535 and miR-1915-5p expression levels in the amniotic fluid serve as clinical predictors of fetal morbidity due to infection [9]. Therefore, analyzing serum miR-4535 and miR-1915-5p expression levels could be informative for the diagnosis of severe chorioamnionitis.

The clinical utility of circulating miRNAs as disease biomarkers has been demonstrated [10]. Recently, the use of circulating miRNAs as diagnostic and prognostic biomarkers of systemic infectious diseases, such as tuberculosis, sepsis, and viral hepatitis, has garnered attention [11]. In severe chorioamnionitis, the fetus is systemically exposed to infectious materials and inflammatory substances in the uterine cavity [1], and this is accompanied by the flow of highly expressed miRNAs from the amniotic fluid into the maternal serum. Thus, miRNA expression in the serum can be used for diagnosing severe chorioamnionitis. Therefore, in this study, we aimed to evaluate the clinical significance of novel biomarkers for severe chorioamnionitis and fetal infection.

## Materials and methods

Histological criteria

Histologically, chorioamnionitis was diagnosed based on acute inflammation in the chorion or amnion [1]. According to Blanc’s classification, in stage III (severe chorioamnionitis), neutrophil infiltration is evident in sub-amniotic connective tissues and the amniotic epithelium; in stage II (chorionitis), neutrophil infiltration is observed in the chorionic plate or membranous chorionic connective tissues; in stage I (sub-chorionitis), mottled or diffuse aggregation of neutrophils is observed in the sub-chorionic plate or deciduous membrane.

Clinical definition

Clinical chorioamnionitis was diagnosed according to the Lencki criteria (maternal fever > 38°C with positive findings on blood investigations, presence of discharge, abdominal pain, or maternal tachycardia) [12]. A high white blood cell (WBC) count (>20,000/μL) or low WBC count (<5000/μL) in the peripheral blood is significantly associated with culture positive for neonatal sepsis [13]. Neonatal C-reactive protein (CRP) and immunoglobulin M (IgM) levels were clinically evaluated to study neonatal sepsis [14]. Considering the diagnostic significance of the WBC counts in neonatal sepsis, in this study, we defined suspected fetal infection as a neonatal WBC count of <5000/μL or >20,000/μL, CRP level of >0.5 mg/mL, or an IgM level of >20 mg/mL after delivery. Medical intervention in newborns was defined as long-term (>5 days) antibiotic administration [9].

Patients

We examined 40 pregnant women at the Department of Obstetrics and Gynecology, Faculty of Medicine, Fukuoka University, Fukuoka, and the Department of Obstetrics and Gynecology, National Hospital Organization Saga Hospital, Saga, Japan, and collected amniotic fluid and serum samples: 11 women with Blanc’s classification [1] stage III (group III), 17 with Blanc’s classification stage II (group II), and 12 with Blanc’s classification stage 0-I (group I). All patients were diagnosed with possible preterm labor, hospitalized, and suspected to have an intrauterine infection. The following patients were excluded: those with multiple pregnancies associated with threatened preterm labor and those who had undergone amniocentesis in the early second trimester.

The clinical results of the newborns were collected for 37 of the 40 pregnant women in all groups; there was one stillborn baby in Group III, and there were two newborns for whom data could not be obtained in Group I. The neonatal peripheral blood sample was collected at birth. This study was approved by the ethics committees of Fukuoka University Hospital and National Hospital Organization Saga National Hospital (approval numbers 15-2-08 and 27-4, respectively). All participants (pregnant women and parents of newborns) provided written informed consent. There has been a very clear explanation of the risks. The clinical and pathologic information of the pregnant women was obtained from medical records.

Sample collection

We performed all methods following the Standards for Reporting of Diagnostic Accuracy Studies (STARD) guidelines and regulations for reporting diagnostic accuracy studies. Amniotic fluid and maternal serum samples were collected from pregnant women between April 1, 2016, and March 31, 2019, at Fukuoka University Hospital (n = 24) and National Hospital Organization Saga National Hospital (n = 16). To collect amniotic fluid samples, amniocentesis was performed percutaneously with guiding transabdominal ultrasound. Amniocentesis was also performed aseptically. Maternal serum samples were collected at the same time as the amniocentesis was performed. Neonatal peripheral blood samples were obtained at birth. Within 24 hours of collection, the amniotic fluid and maternal and neonatal serum samples were centrifuged at 2400 x g for 20 min at 25°C. The supernatant was stored at −80°C for RNA extraction.

Total RNA extraction

Total RNA was extracted from each amniotic fluid and serum sample using the miRNeasy serum/plasma kit (Qiagen, Hilden, Germany). Subsequently, 200 μL of the amniotic fluid or serum supernatant was mixed with 1.0 mL of QIAzol lysis reagent (Qiagen, Hilden, Germany), and the samples were reacted at 25°C ± 1°C for five minutes. In the samples of the control, 3.0 μL of a commercially available synthetic Caenorhabditis elegans miR-39-3p (6.0 × 10¹² copies/μL, Cel-miR-39-3p, Genenet Co., Ltd., Fukuoka, Japan) was added to each sample. The adjusted total RNA was then isolated in accordance with the protocol specified by the manufacturer. Each sample was fully eluted in 35 μL of RNase-free water (Qiagen, Hilden, Germany) and stored at −80°C.

Reverse transcription and digital droplet polymerase chain reaction (ddPCR)

A total of 5 mL of RNA was reacted with primers specific for both miRNA and Cel-miR-39-3p, as included in the TaqMan™ MicroRNA Reverse Transcription Kit (Applied Biosystems, Foster City, CA, USA), to synthesize cDNA. Quantification of miRNA expression was conducted using a ddPCR kit (Bio-Rad Laboratories, Hercules, CA, USA) and a TaqMan miRNA assay (Applied Biosystems, Foster City, CA, USA). Specifically, 2 μL of cDNA was combined with 18 μL of QX200 reagent (Bio-Rad Laboratories) in accordance with the protocol specified by the manufacturer, and the resulting mixture was partitioned into approximately 20,000 droplets using a QX200 droplet generator (Bio-Rad Laboratories). Polymerase chain reaction (PCR) was conducted in accordance with the following parameters: an initial incubation period of five minutes at 95°C, followed by 40 cycles of 30 seconds at 95°C and one minute at 52°C, and finally an incubation period of five minutes at 4°C and 90°C, respectively. The temperature of the PCR was decreased at a rate of 2°C per second to reach a final temperature of 4°C (annealing temperature, 52°C). Fluorescence analysis was conducted using a QX200 Droplet Reader (Bio-Rad Laboratories), and the copy number of miRNAs in 1 mL of sample was quantified using Bio-Rad QuantaSoft v1.7 (Bio-Rad Laboratories). The expression of miRNAs was normalized to the expression of the synthetic spike-in miR-39-3p. TaqMan primers and probes for each miRNA were obtained from Applied Biosystems (Foster City, CA, USA).

Statistical analysis

Kruskal-Wallis test was conducted to determine statistical significance in multi-group comparisons, whereas Dunn’s multiple comparison test was used for pairwise comparisons. All experiments were conducted in triplicate, and the data are expressed as mean ± interquartile range. Fisher's exact test was used to determine statistical significance, and the data are shown as n (%). A correlation was determined based on the Pearson correlation coefficient (r). Receiver operating characteristic (ROC) curves were constructed to evaluate the diagnostic significance of fetal infection. We determined the AUC, cut-off value, and Youden indices. In addition, categorical variables were analyzed using Fisher's exact test to determine statistical significance. Statistical significance was set at *P* < 0.05. All statistical analyses were performed using GraphPad Prism v9.0 (GraphPad Software, San Diego, CA, USA) and SPSS v16.0J (SPSS Japan, Tokyo, Japan) for Windows.

## Results

Assessment of the study participants

Demographic and clinical characteristics were obtained by a chart review (Table 1).

**Table 1:** Maternal and neonatal characteristics in Blanc’s classification groups. † data are shown as median (range). § data are shown as n (%). * indicates a significant difference (P < 0.05). “-” indicates data not available. WBC: white blood cell; CRP: C-reactive protein; IgM: immunoglobulin M

-	(A) group III	(B) group II	(C) group I	P-value
Maternal characteristics at amniocentesis	(n = 11)	(n = 17)	(n = 12)	-
Gestational age at amniocentesis (weeks) †	28.5 (25.8–31.1)	1.3 (24.3–38.3)	34.5 (28.3–40.4)	0.0028*
Body temperature > 38.0 (°C) §	2 (18.2)	0 (0.0)	0 (0.0)	0.0623
Heart rate > 100 (/min) §	5 (45.5)	3 (17.7)	2 (16.7)	0.1837
Tenderness of the uterus§	0 (0.0)	0 (0.0)	0 (0.0)	1.000
Stench of vaginal discharge or amniotic fluid §	0 (0.0)	0 (0.0)	0 (0.0)	1.000
WBC count in maternal peripheral blood > 15,000 (cells/μL) §	7 (63.6)	6 (35.3)	0 (0.0)	0.0047*
CRP in maternal peripheral blood (mg/dL) †	2.5 (0.7–12.7)	1.9 (0.1–9.4)	0.8 (0.02–6.3)	0.043*
Neonatal characteristics after birth	(n = 10)	(n = 17)	(n = 10)	-
Gestational age at birth (weeks) †	28.5 (26.0–31.6)	33.3 (25.7–38.3)	34.5 (28.3–40.4)	0.0076*
Neonatal body weight (g) †	1118 (838–1495)	1967 (777–2411)	1795 (1203–2473)	0.0126*
Apgar score 1 min †	7.0 (1–9)	7.0 (4–9)	8.0 (1–8)	0.5936
Apgar score 5 min †	8.0 (5–9)	8.0 (6–9)	9.0 (3–9)	0.2138
Umbilical arterial pH †	7.389 (7.23–7.44)	7.406 (7.29–7.51)	7.299 (7.12–7.49)	0.1672
WBC count in neonatal peripheral blood (cells/μL) †	1.14 × 10^4^ (4.1 × 10^3^–2.4 × 10^4^)	1.12 × 10^4^ (3.8 × 10^3^–5.9 × 10^4^)	1.04 × 10^4^ (6.2 × 10^3^–2.3 × 10^4^)	0.8914
CRP in neonatal peripheral blood (mg/dL) †	0.20 (0.0–1.6)	0.01 (0.0–1.9)	0.02 (0.0–0.6)	0.016*
IgM in neonatal peripheral blood (mg/dL) †	11.0 (6–68)	6.0 (0–24)	6.0 (4–13)	0.035*

These groups were categorized according to Blanc’s classification [1], with Groups I-III comprising patients with Blanc’s classification stage 0-I (sub-chorionitis or non-infection), stage II (chorionitis), and stage III disease (chorioamnionitis), respectively, as described in detail in the Materials and Methods section.

Maternal WBC counts were significantly higher in Groups II and III than in Group I (*P* = 0.0477). Neonates in Group III had significantly younger gestational ages at birth (weeks) than those in Group I (*P* = 0.0076). Moreover, neonatal body weight was significantly lower in group III than in Groups I and II (*P* = 0.0126). CRP and IgM levels in neonatal peripheral blood were significantly higher in Group III than in Groups I and II (*P* = 0.016 and 0.035, respectively).

Relationship of Serum miRNAs With Histological Chorioamnionitis and Amniotic Fluid miRNA Expression

**Figure 1:**
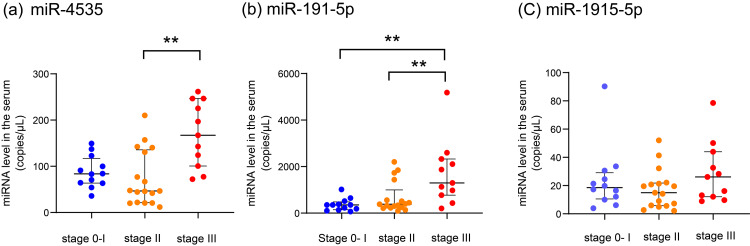
Relationship of serum miRNAs with histological chorioamnionitis: (a) miR-4535, (b) miR-191-5p, and (c) miR-1915-5p levels and Blanc’s classification groups. Blue, orange, and red circles indicate the miRNA copy number in Blanc’s classification stages 0–I, II, and III, respectively. The thick bar indicates the median, and the thin bars indicate the 25th percentile and 75th percentile, respectively. ** P < 0.01.

To confirm whether serum miRNAs are suggestive of histological chorioamnionitis, we examined the association between Blanc’s classification and serum miRNA expression (Fig. 1).

The serum miR-4535 level was significantly higher in Group III than in Group II (*P* = 0.0014). The serum miR-191-5p level was significantly higher in Group III than in Groups I and II (*P* = 0.031), but was not significantly different between Groups I and II (*P* = 0.2586).

**Figure 2:**
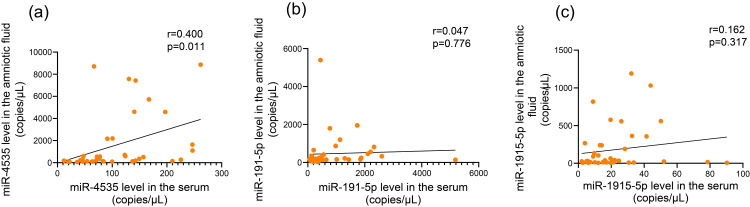
Correlation between serum and amniotic miRNA expression levels: (a) miR-4535, (b) miR-191-5p, and (c) miR-1915-5p levels. The horizontal and vertical axes indicate serum and amniotic miRNA levels, respectively. r indicates the correlation index.

We investigated the relationship between serum and amniotic fluid miRNA levels and found that only miR-4535 exhibited a significant correlation between serum and amniotic fluid levels in all patients (r = 0.400, *P* = 0.011; Fig. 2).

**Figure 3:**
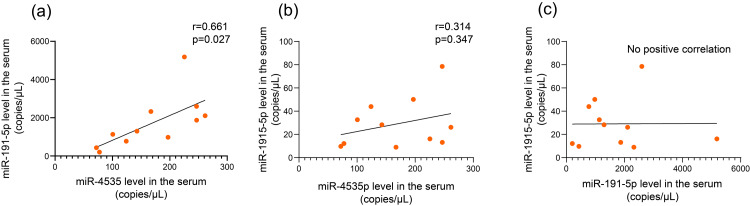
Correlation between serum (a) miR-4535, (b) miR-191-5p, and (c) miR-1915-5p levels in Group III. The horizontal axis indicates serum (a) miR-4535, (b) miR-4535, and (c) miR-191-5p (c) levels. The vertical axis indicates serum (a) miR-191-5p, (b) miR-1915-5p, and (c) miR-1915-5p levels. r indicates the correlation index.

Moreover, the serum miR-4535 and miR-191-5p levels significantly correlated in Group III (r = 0.661, *P* = 0.027). However, no significant correlations were observed in the other combinations (Fig. 3).

Diagnostic Significance of Serum miRNA Levels for Chorioamnionitis

To predict severe chorioamnionitis based on serum miR-4535, miR-191-5p, and miR-1915-5p expression levels, we calculated the AUC, cut-off value, Youden index, and sensitivity and specificity of the ROC curve in Group III (Table 2).

**Table 2:** Diagnostic accuracies of miRNA levels in serum samples for Blanc’s classification stage III. “-” indicates data not available. AUC; area under the curve

-	AUC	Cut-off value	Youden Index	Sensitivity	Specificity
hsa-miR-45re	0.864	71.3 copies/mL	0.567	1.000	0.567
hsa-miR-191-5p	0.836	712.3 copies/mL	0.652	0.818	0.833
hsa-miR-1915-5p	0.645	25.4 copies/mL	0.346	0.545	0.800

The AUC for serum miR-4535 and miR-191-5p expression levels indicated a high diagnostic accuracy for chorioamnionitis ((miR-4535: 0.8649 (asymptotic 95% confidence interval (CI): 0.7458-0.9815), miR-191-5p; 0.836 (asymptotic 95% CI: 0.6865-0.9863)). We then compared the diagnostic accuracy of serum chorioamnionitis miRNA, Lencki criteria (maternal fever, maternal tachycardia, and WBC count), and CRP in all groups. The miR-4535 level, miR-191-5p level, maternal WBC count, and CRP level were significantly higher in Group III than in the other groups (Table 3).

**Table 3:** Association between Blanc’s classification with the levels of mir-4535, mir-191-5p, and mir-1915-5p, as well as the body temperature, heart rate, WBC count in maternal peripheral blood, and CRP level in maternal peripheral blood at the time of amniocentesis. * indicates a significant difference (P < 0.05). “-” indicates data not available. ‡ data are shown as n (%). WBC; white blood cell, CRP; C-reactive protein

-	Blanc III (n = 11)	Blanc 0-II (n = 29)	P-value Fisher's exact test
miR-4535 (copies/μL)	-	-	-
>71.3	11 (100) ^‡^	13 (45)^ ‡^	0.0012*
<71.3	0 (0) ^‡^	16 (55)^ ‡^	
miR-191-5p (copies/μL)	-	-	-
>712.3	9 (82) ^‡^	5 (17) ^‡^	0.0003*
<712.3	2 (18) ^‡^	24 (83) ^‡^	
miR-1915-5p (copies/μL)	-	-	-
>25.4	6 (55) ^‡^	6 (21) ^‡^	0.0563
<25.4	5 (45) ^‡^	23 (79) ^‡^	
Body temperature (°C)	-	-	-
>38.0	2 (18) ^‡^	0 (0) ^‡^	0.0705
<38.0	9 (82) ^‡^	29 (100) ^‡^	
Heart rate (/min)	-	-	-
>100	5 (45) ^‡^	5 (17) ^‡^	0.1028
<100	6 (55) ^‡^	24 (83) ^‡^	
WBC count in maternal peripheral blood (cells/μL)	-	-	-
>15,000	7 (63) ^‡^	6 (21) ^‡^	0.0204*
<15,000	4 (36) ^‡^	23 (79) ^‡^	
CRP level in maternal peripheral blood (mg/dL)	-	-	-
>2.0	9 (82) ^‡^	11 (38) ^‡^	0.031*
<2.0	2 (18) ^‡^	18 (62) ^‡^	

Clinical Significance of miRNAs for Neonatal Infection

To investigate the relationship between neonatal inflammation and maternal serum miRNA levels, we compared the expression levels of miR-4535, miR-191-5p, and miR-1915-5p expression levels in the presence of abnormal findings, including neonatal WBC count and CRP and IgM levels, among all groups (Table 4).

**Table 4:** Diagnosis accuracy for neonatal infection (neonatal white blood cell count <5,000 or >20,000 cells/µL, neonatal CRP, and IgM positive after birth). “-” indicates data not available. AUC; area under the curve, WBC; white blood cell, CRP; C-reactive protein, IgM; Immunoglobulin M

-	AUC	Cut-off value	Youden Index	Sensitivity	Specificity
WBC count <5,000 or >20,000 cells/µL	-	-	-	-	-
miR-4535 (copies/μL)	0.655	141.4	0.377	0.556	0.821
miR-191-5p (copies/μL)	0.603	439.1	0.385	0.778	0.607
miR-1915-5p (copies/μL)	0.325	3.3	0.071	1.000	0.071
Neonatal CRP level >0.5 (mg/dL)	-	-	-	-	-
miR-4535 (copies/μL)	0.636	71.3	0.424	1.000	0.424
miR-191-5p (copies/μL)	0.674	414.9	0.545	1.000	0.545
miR-1915-5p (copies/μL)	0.447	8.1	0.242	1.000	0.242
Neonatal IgM level ≥20 (mg/dL)	-	-	-	-	-
miR-4535 (copies/μL)	0.922	153.2	0.882	1.000	0.882
miR-191-5p (copies/μL)	0.745	536.8	0.647	1.000	0.647
miR-1915-5p (copies/μL)	0.657	12.6	0.412	1.000	0.412

Based on the ROC curve, miR-4535 demonstrated high diagnostic accuracy for patients with IgM levels> 20 mg/mL (AUC: 0.922 (asymptotic 95% CI: 0.8526-1.007)). Similarly, the AUC for miR-4535 exhibited higher accuracy than that of the maternal WBC count or CRP level for patients using antibiotics for a long term (five days) (miR-4535: 0.805 (asymptotic 95% CI: 0.6657-0.9435), WBC count: 0.542 (asymptotic 95% CI: 0.3270-0.7563), and CRP level: 0.644 (asymptotic 95% CI: 0.4662-0.8211) (Table 5).

**Table 5:** Diagnosis accuracy of long-term administration of antibiotics in newborns for fetal morbidity due to infection. “-” indicates data not available. AUC; area under the curve, WBC; white blood cell, CRP; C-reactive protein

-	AUC	Cut-off value	Youden Index	Sensitivity	Specificity
miR-4535 (copies/μL)	0.805	87.62	0.579	0.690	0.889
miR-191-5p (copies/μL)	0.747	414.9	0.544	0.655	0.889
miR-1915-5p (copies/μL)	0.517	21.4	0.134	0.690	0.444
Maternal WBC count (count/μL)	0.542	12,900	0.238	0.571	0.667
Maternal CRP level (mg/dl)	0.644	2.250	0.468	0.690	0.778

## Discussion

In this study, significantly high serum miR-4535 and miR-191-5p expressions were observed in pregnant women with severe chorioamnionitis. Moreover, the AUC values for miR-4535 and miR-191-5p indicated their good diagnostic accuracy for severe chorioamnionitis. Our findings suggest that the more severe the chorioamnionitis, the higher the serum levels of miR-191-5p and miR-4535. In addition, we observed a significant association between miR-4335 expression in the serum and amniotic fluid of pregnant women without membrane rupture. However, only the AUC for miR-4535 indicated good accuracy for elevated neonatal IgM levels and fetal morbidity due to infection. These results suggest that serum miR-4535 is more sensitive than other inflammatory markers.

Interleukin (IL-6), CRP, amniotic neutrophil elastase, blood, and vaginal flora serve as biomarkers of chorioamnionitis. Romero et al. reported that the level of IL-6 in the amniotic fluid serves as an indicator of intrauterine infection based on amniotic fluid culture results (AUC = 0.927) [15]. In our study, the maternal WBC count and CRP level, combined with glucose level, cell count, and lactate dehydrogenase level in the amniotic fluid, had an AUC value, sensitivity, and specificity of 0.803, 87.5%, and 74.0%, respectively [16]. In a previous study, vaginal flora analysis showed an AUC value, sensitivity, and specificity of 0.849, 71.4%, and 82.4%, respectively [17]. In the present study, the AUC, sensitivity, and specificity were 0.864, 100%, and 57%, respectively, for serum miR-4535 and 0.836, 81%, and 83%, respectively, for serum miR-191-5p. In our previous study, these parameters were 0.737, 67.5%, and 76.5%, respectively, for amniotic miR-4535; 0.649, 82.5%, and 52.9%, respectively, for amniotic 16S rDNA; and 0.664, 57.5%, and 76.5%, respectively, for amniotic IL-6 to predict fetal morbidity due to infection [9]. This study revealed AUC, sensitivity, and specificity of 0.805, 69.0%, and 88.9%, respectively, for serum miR-4535. These findings suggest that for neonatal infection, the AUC, sensitivity, and specificity of 16S rDNA, IL-6, and miR-4535 in the amniotic fluid are similar [9]. Serum miR-4535 measurement is a minimally invasive procedure for the diagnosis of chorioamnionitis and neonatal infection, as blood tests are less invasive and easier to perform than amniocentesis. Therefore, serum miR-4535 and miR-191-5p are considered favorable serum biomarkers for chorioamnionitis.

miRNAs are non-coding RNAs that regulate the expression of target genes and are associated with cell proliferation and differentiation. In vivo, miRNAs are considered useful biomarkers for systemic diseases such as infectious diseases and malignant tumors. Several miRNAs are associated with chorioamnionitis. microRNA-548 is involved in the etiology of preterm labor by altering inflammatory responses and regulating high mobility group box 1 protein expression in human amniotic epithelial cells [18]. Membranes with chorioamnionitis display increased miR-223 and miR-338 expression, and miR-223 target genes (*SPINK5*, *TGFB2*, and* IFNB1*) are involved in the biological processes of negative regulation of immune and anti-inflammatory responses [19]. Furthermore, miR-518b and miR-338-3p are differentially expressed in acute and non-acute chorioamnionitis, and placental miR-518b expression is associated with IL-6 SNP (single-nucleotide polymorphism), which is closely related to several inflammatory disorders [20]. Although amniotic miRNA expression in chorioamnionitis has not been reported, reports of miRNA expression in the placenta and placental membranes suggest that alterations in amniotic miRNA expression may be useful in predicting chorioamnionitis [18].

Circulating miRNAs, which protect against RNase, are delivered as RNA-binding proteins and lipoprotein complexes or as extracellular vesicles (EVs) [11,21]. EVs comprise bioactive molecules, such as nucleic acids, metabolites, proteins, and pharmacological compounds [11,21]. Almost all cell types, including cancer, stem, and inflammatory cells, secrete EVs into the extracellular space [21]. EVs can be divided into three classes based on their size: exosomes (approximately 50-150 nm in diameter), microvesicles (approximately 100-1000 nm in diameter), and apoptotic bodies (approximately 500-2000 nm in diameter) [21]. In advanced cancer, exosomes are abundantly secreted in effusions into cavities, such as in ascites, and pulmonary effusion. Notably, the profiles of peripheral circulating miRNAs are similar to those of miRNAs in ascites and pulmonary effusion [22]. In the present study, the serum miR-4535 level, which was approximately less than one-tenth of that in the amniotic fluid, corresponded to that in the amniotic fluid of pregnant women with Blanc’s classification stage 0-III disease. This finding suggests that excess specific protein-binding miRNAs or exosomal miRNAs in the amniotic fluid may be effused in a manner akin to that of circulating miRNAs in patients with severe chorioamnionitis. Recently, miR-4535 was shown to downregulate TGF-b/smad4 signaling in fibroblasts or inactivate the autophagy pathway in melanoma stem cells [23,24]. It alleviates UVB-induced skin photodamage by promoting TGFβ/Smad collagen synthesis signaling, reducing epidermal hyperplasia, wrinkle formation, and skin senescence, and inhibiting hsa-miR-4535 expression [23]. Upon entering melanoma parental cells (MPCs), miR-4535 enhances the metastatic colonization of MPCs by deactivating the autophagy pathway [24]. Overall, these findings suggest that miR-4535 may be involved in oxidative damage. Therefore, upregulated miR-4535 expression, such as that in a cytokine storm, may be indicative of cellular stress or damage.

The target genes of miR-4535 include *CD44*, *LHFPL3*, *KDM1B*, *RNF19A*, *C19orf82*, and *FKBP4* [8]. CD44 is involved in cell adhesion and migration during various life events, such as organogenesis, neuroaxonogenesis, hematopoiesis, and wound healing. It binds to hyaluronic acid, a component of the extracellular matrix present in vascular endothelial cells, and is involved in cell proliferation and migration as a cell adhesion molecule during inflammation. As CD44 is involved in lymphocyte rolling, elevated miR-4535 levels may represent a lymphocyte migration response during intrauterine infection. By contrast, CD44 deficiency regulates the expression of many genes involved in p53 signaling, apoptosis, and autophagy, resulting in the inhibition of apoptosis and induction of autophagy during *Edwardsiella piscicida *infection [25]. Notably, CD44 variants exert protective effects against *E. piscicida* infection in zebrafish, indicating the critical role of CD44 in mediating a protective immune response against *E. piscicida*. Therefore, the increased expression of miR-4535, a target gene of CD44, may represent a disruption in the defense mechanism against bacterial infection in utero owing to the decreased expression of its target gene, *CD44*.

In the present study, miR-191-5p was associated with severe chorioamnionitis, although no relationship was observed between miR-191-5p expression in the serum and amniotic fluid of pregnant women according to Blanc’s classification of stages 0-III. miR-191-5p, one of the six circulating miRNAs, has demonstrated effective discrimination in severe sepsis and may play a pivotal role in the anti-inflammatory response, leading to the suppression of immune cell activation in severe sepsis and inflammation [26]. In addition, miR-191-5p transfection in rat models suppresses septic acute kidney injury by targeting oxidative-stress responsive 1, suggesting that circulating miR-191-5p upregulation may contribute to the protection of major organs, such as the kidney, from inflammatory damage [27]. Amyloid-b_1-40_ mediates the damage of retinal pigment epithelium by decreasing endogenous miR-191-5p expression, and miR-191-5p overexpression ameliorates cell injury in the retinal pigment epithelium by suppressing inflammation [28]. Accordingly, studies have focused on the function of miR-191-5p, highlighting its potential antioxidant or anti-inflammatory roles in inflammatory disorders [26-28]. In pregnant women with severe chorioamnionitis, upregulation of circulating miR-191-5p expression may compensate for inflammatory damage to maternal organs.

In the present study, the miR-4535 and miR-1915-5p levels in the serum were less than a tenth or hundredth of those in the amniotic fluid of pregnant women with severe chorioamnionitis, suggesting that excess miR-4535 and miR-1915-5p in the amniotic fluid may flow into the blood as circulating miRNAs. These results suggest that miR-4535 and miR-1915-5p may originate from fetoplacental tissues. By contrast, the serum and amniotic miR-191-5p levels were slightly different. Circulating miR-191-5p originates from the immune system [25,29,30]. Accordingly, miR-1915-5p may originate from the maternal immune cells in pregnant women with severe chorioamnionitis. Based on this evidence, miR-191-5p can be regarded as a reliable biomarker for maternal infections in severe chorioamnionitis, whereas miR-4535 is a promising biomarker for both maternal and fetal infections in severe chorioamnionitis.

There is a lively debate about the accuracy of the diagnosis of chorioamnionitis and the timing of therapeutic intervention and pregnancy termination, with a variety of opinions. These miRNAs can accurately predict chorioamnionitis and neonatal infection during pregnancy. Most notably, this diagnostic method does not require amniocentesis and can use maternal peripheral blood. Some pregnant women who have few clinical symptoms may refuse to undergo amniocentesis. Still, maternal peripheral blood samples can greatly reduce the testing hurdle, facilitating early prediction of chorioamnionitis and neonatal infections. Early prediction of chorioamnionitis during pregnancy will enable prompt referral of a patient for antimicrobial therapy or pregnancy termination.

However, the physiological function of miRNAs during pregnancy remains unclear. Moreover. the present study was limited by its retrospective nature and small number of patients. Hence, further investigations are required to identify the cell types expressing miR-4535 in fetal appendages and placental tissues and examine the behavior of cells transfected with miR-4535 or shRNA-4535 in vitro. Nevertheless, miR-4535 is an intronic miRNA with no other gene family members and has been rarely reported to date. Therefore, to determine the mechanisms by which miR-4535 affects infection and inflammation in chorioamnionitis and immune function, comprehensive studies involving a larger cohort of patients are required. Moreover, the expression of miR-4535 should be validated in the umbilical cord blood and blood of newborns.

## Conclusions

The diagnosis of chorioamnionitis is challenged by the difficulties in predicting the disease during pregnancy and the hurdles involved in amniocentesis. In this study, we found that miR-4535 in the serum may be involved in severe chorioamnionitis and fetal morbidity of infection, whereas miR-191-5p may be linked to maternal infection. However, for their practical application, further research is needed to clarify the mechanisms by which miR-4535 affects infection and inflammation in chorioamnionitis and immune function. Confirmation of the expression of each miR in the placenta may help elucidate this mechanism. Future research in this direction may aid in the development of diagnostic and therapeutic approaches for severe chorioamnionitis using noninvasive methods without amniocentesis; it would also aid in reducing neonatal infections and improving neonatal outcomes.
